# Comparison of transepithelial and conventional photorefractive keratectomy in myopic and myopic astigmatism patients: a randomized contralateral trial

**DOI:** 10.1186/s12886-022-02293-2

**Published:** 2022-02-11

**Authors:** Hassan Hashemi, Azam Alvani, Mohammadreza Aghamirsalim, Mohammad Miraftab, Soheila Asgari

**Affiliations:** 1 Noor Ophthalmology Research Center, Noor Eye Hospital, 96, Esfandiar Blvd, Valiasr St, Tehran, Tehran 1968653111 Iran; 2 Translational Ophthalmology Research Center, Tehran University of Medical Sciences, Tehran, Iran

**Keywords:** Transepithelial photorefractive keratectomy, mechanical epithelial debridement photorefractive keratectomy, alcohol-assisted photorefractive keratectomy, corneal epithelial defect area, corneal epithelial healing rate

## Abstract

**Background:**

To assess transepithelial photorefractive keratectomy (tPRK) in terms of corneal epithelial healing rate, postoperative pain, postoperative discomfort, and visual and refraction outcomes compared to mechanical epithelial debridement PRK (mPRK) and alcohol-assisted PRK (aaPRK).

**Methods:**

In this double-masked, randomized clinical trial, thirty-nine patients underwent tPRK in one eye and mPRK in the fellow eye (arm A), and 33 patients underwent tPRK in one eye and aaPRK in the contralateral eye (arm B). All surgical procedures were done using the Schwind Amaris excimer laser. The area of corneal epithelial defect in all eyes was captured and analyzed using ImageJ software.

**Results:**

Mean epithelial healing time was respectively 3.74 ± 0.82 and 3.59 ± 0.79 days in tPRK versus mPRK (*P* = 0.21) in arm A, and 3.67 ± 0.92 and 3.67 ± 0.74 days in tPRK versus aaPRK (*P* = 1.00) in arm B. Accounting for the initial corneal epithelial defect area, the epithelial healing rate was faster in conventional PRK groups compared to tPRK (both *P*<0.001) in both arms. However, there was no significant difference in safety, efficacy, spherical equivalent refractive accuracy, or corneal haze development between tPRK and conventional PRK groups (all *P* > 0.05).

**Conclusions:**

All three methods are effective in terms of visual and refractive outcomes. However, although time to complete re-epithelialization was similar with the three methods, the epithelial healing rate was faster in conventional PRK considering the initial corneal epithelial defect area, and the patients experienced less pain and discomfort in the first postoperative day.

**Trial registration:**

IRCT, IRCT20200317046804N1. Retrospectively registered 5 May 2020.

## Introduction

Photorefractive keratectomy (PRK) is an effective surface ablation procedure for the correction of myopia and myopic astigmatism [1]. This procedure involves the removal of the epithelium followed by laser ablation of Bowman’s layer and the anterior stroma to remodel the cornea and correct ametropia. Presence of epithelial defect in PRK results in pain, discomfort and slow visual recovery in the early postoperative period. Another undesirable complication of PRK is the development of corneal stromal haze, which is attributed to basement membrane damage and the reconstruction of stromal tissue post-operation [2, 3]. Previous studies have indicated that the severity of corneal haze is affected by the time to re-epithelialization of the treated corneal surface [4]. Therefore, a quicker epithelial healing process is an important contributor to complete corneal regeneration and successful outcomes. Faster epithelial healing also accelerates visual recovery and relief from pain and discomfort, allows patients to return to their daily activities sooner, and decreases the risk of adverse events.

In addition to the amount of refractive errors, postoperative inflammatory responses, and genetic factors, epithelial healing speed may be influenced by the surgical procedure [5, 6]. Different epithelial removal methods have been developed to try to overcome the disadvantages of PRK, including conventional [mechanical epithelial debridement (mPRK) and alcohol-assisted (aaPRK)] and excimer laser [transepithelial (tPRK)] methods [7, 8]. tPRK is one of the newest methods of PRK in which the epithelium and stroma are ablated in one step without the use of any mechanical tools or chemicals [9]. Since there is no interval between epithelial and stromal ablations, this method is expected to be associated with a faster surgical time and a lower chance of stromal dehydration during surgery compared to conventional PRK methods [9, 10]. Moreover, since the diameter of the corneal epithelial defect (CED) is smaller in tPRK, faster healing and less pain and discomfort shortly after surgery are expected [9, 10]. Several studies have compared tPRK with mPRK and aaPRK [10–17]. Although all studies have shown that the three methods are effective in terms of visual outcomes, reports regarding postoperative pain, discomfort, and epithelial healing time are inconclusive, which is mainly due to lack of standard protocols for CED size measurement and calculation of healing rate.

This contralateral clinical trial was designed to compare cases receiving tPRK in one eye and mPRK or aaPRK in the fellow eye. Epithelial healing rate was assessed using consecutive daily photographs of the CED starting from the day of surgery and accurate calculation and monitoring of the CED area. Moreover, the intensity of pain and other symptoms in the early postoperative days and the visual and refraction outcomes three months after surgery were compared between tPRK and conventional PRK methods.

## Methods

### Patients and sampling

This double-masked, randomized contralateral clinical trial was conducted on myopic and myopic astigmatism patients scheduled for PRK at Noor Eye Hospital, Tehran, Iran in 2020 based on good clinical practice principles. Inclusion criteria were age 18–50 years, myopia of −2.00 to −8.00 diopters (D), astigmatism of −4.00 D or less, a stable refraction for at least one year, and contact lens wear abstinence for three weeks. The exclusion criteria were any ocular pathology, corneal dystrophy, keratoconus or keratoconus suspect, glaucoma or glaucoma suspect, diabetes, autoimmune diseases, and history of ocular surgery.

### Ethical considerations

This study adheres to CONSORT 2010 guidelines. The tenets of the Helsinki Declaration were observed in all stages of the study. The protocol of this study was approved by the Ethics Committee of Tehran University of Medical Sciences; it is registered and publicly accessible at: https://www.irct.ir/search/result?query=IRCT20200317046804N1.

Written informed consent was obtained from all participants prior to enrollment.

### Randomization, concealment, and masking

Randomization was done in two stages. First, each patient was randomly assigned to one of the two study arms (A and B). In the second stage, randomization was done to allocate fellow eyes of each patient to a type of treatment. In arm A, the randomly selected eye received tPRK and the fellow eye received mPRK. In arm B, the randomly selected eye received tPRK and the contralateral eye received aaPRK. Balanced block randomization with a block size of four was done in both stages to allocate the patients to arms or eyes to treatment. The random sequence of blocks was obtained from the random number table. To maintain concealment, randomization was done by a person other than the surgeon using sequentially numbered, sealed, opaque envelopes. The patients and assessing technicians were unaware of the allocation of eyes until the end of the study, and the surgeon was blind to allocation until surgery.

### Surgical technique

All procedures were done using the Schwind Amaris excimer laser (SCHWIND eye-tech-solutions GmbH & Co KG) by the same surgeon (H.H.). The target refraction was emmetropia for all eyes. After controlling the fixation and head position by the surgeon and the Schwind system, first the left eye and then the right eye received the allocated treatment.

In mPRK, the cornea was marked with an 8.5 mm marker (B.S.A.Co.) and the epithelium was removed using a hockey-stick spatula. In aaPRK, a 8.5 mm well was placed on the central cornea and filled with ethanol 20% (Sina Daru). After 30 s, the alcohol was absorbed using a small dry polyvinyl alcohol sponge (Merocel, Medtronic, Inc.) and the loose epithelium was removed from the cornea. In both conventional methods, the stroma was ablated with laser immediately after epithelial debridement.

For tPRK, ORK-CAM software module (SCHWIND eye-tech- solutions GmbH & Co KG) was used for automatic measurement of the amount of corneal ablation. In single-step tPRK, first, the cornea is ablated for refractive correction, then, additional ablation is done to compensate for the epithelial thickness (software default is 55 μm).

The optical zone (OZ) was set to 6.5 mm for all eyes. Laser was applied at a frequency of 1050 Hz and wavelength of 193 nm. To reduce patients’ anxiety, the horizontal diameter of the cornea (white to white) was measured using a Castroviejo caliper (B#8232, B.S.A.Co.) after the ablation was over. Mitomycin C (MMC) 0.02% was mixed with fluorescein and applied to the stromal surface for 5 s per one diopter correction using a sponge. The image of the ablated cornea was captured under the cobalt blue light using the camera mounted to the excimer laser machine. Then, the ocular surface was washed with copious amounts of balanced salt solution (BSS). After instilling one drop of chloramphenicol (Sina Daru), a bandage contact lens (Ciba vision) was placed on the cornea. The epithelial removal time in mPRK and aaPRK was measured with a stopwatch. For all cases of tPRK, the epithelial removal time was 13 s as indicated by Schwind’s software.

The postoperative regimen for all patients included betamethasone 0.1% (Sina Daru) and chloramphenicol 0.1% (Sina Daru) eye drops every 6 h and preservative-free artificial tears (Artelac Advanced, Bausch & Lomb) every three hours. Chloramphenicol was discontinued two days after bandage contact lens removal. Betamethasone was continued for two weeks after the operation; thereafter, fluorometholone 0.1% (Sina Daru) was administered every 6 h and tapered over the next three months considering the amount of stromal ablation.

### Pre and postoperative assessment

Before the operation, uncorrected and corrected distance visual acuity (UDVA and CDVA) were measured using the Snellen SC-2000 chart (Nidek Inc.) and manifest and cycloplegic refraction were measured using retinoscopy (ParaStop HEINE BETA 200; HEINE Optotechnik) for all patients. They also received a complete ophthalmic examination using slit lamp (Haag-Streit), the Goldmann applanation tonometer mounted on the slit lamp, and the Pentacam HR (Oculus Optikgeräte GmbH).

For epithelial healing assessment, the patients were examined daily by an ophthalmologist until complete healing was achieved. In each visit, slit lamp biomicroscopy was done to assess the CED, corneal transparency, presence of filamentary keratitis, and other complications. For accurate measurement of the CED area, the bandage lens was removed and the cornea was photographed using the Photo-Slit Lamp BX 900 (Haag-Streit) after dying the cornea with fluorescein. Then, a new bandage lens was fitted. After complete re-epithelialization, the bandage lens was removed.

The CED area was calculated using the ImageJ software. Each image was imported into the software and the white-to-white corneal diameter was set on the image as the reference distance. The CED border was marked using a cursor, and its area was calculated automatically by the software.

Pain intensity and other symptoms were recorded at each visit by an interviewer blinded to treatment allocation. An 11-point numerical scale was applied to rate the severity of pain, foreign body sensation, burning, photophobia, dry eye, and tearing with 0 indicating no complaints and 10 indicating maximum complaints. The patient was asked about the presence of each symptom in either eye. If the answer was positive, further questions were asked to determine the eye with more severe symptoms. Then, the patient was requested to rate the severity of the symptom in each eye. The 11-point numerical scale was again administered on the 7th postoperative day through a telephone interview.

The patients were evaluated for visual and refraction outcomes and haze development three months after the operation. Corneal haze was assessed using slit lamp biomicroscopy and graded subjectively according to the method suggested by Fantes et al. [18] as follows: 0 = clear cornea; 0.5 = trace haze seen only by oblique illumination; 1 = minimal cloudiness barely seen with direct and diffuse illumination; 2 = mild easily visible opacity; 3 = moderate dense opacity that partially interferes with iris details; 4 = opaque cornea.

### Statistical analysis

The sample size was determined based on the mean and standard deviation of the CED size in tPRK and mPRK groups in the third postoperative day in a contralateral eye study [11]. Using 0.56 ± 0.99 and 2.54 ± 1.01 mm for the tPRK and mPRK groups, respectively, and a type I error of 5% (two sided), 99% power, and tPRK-to-mPRK groups ratio of 1:1, a sample size of 10 in each group was calculated. Given that the main purpose of this study was to compare CED size in the first three postoperative days, and since the difference between the two groups in the first and second days after surgery may not be significant, a sample size of 40 in each group was considered. The Stata statistical software: Release 14 (Stata, Corp LP, College Station) and R package version 3.5.2 were used for data analysis. The Q-Q plot was applied to assess the distribution of variables. In each arm, paired t test was used to compare pre- and postoperative visual acuity and refraction, epithelial removal time, subjective symptoms, and corneal haze between tPRK and conventional methods (mPRK and aaPRK).

Qualitative variables were compared using chi square and Fisher’s exact tests. Safety was assessed based on CDVA changes after surgery compared to baseline, and the safety index was calculated as the mean postoperative CDVA divided by the mean preoperative CDVA. The efficacy index was calculated as the mean postoperative UDVA divided by the mean preoperative CDVA. Generalized estimating equations (GEE) were used to compare safety and efficacy indexes between the study groups while controlling the correlation between fellow eyes (unstructured correlation matrix). Accuracy was assessed by comparing the three-month spherical equivalent refractive errors with the intended target refraction. A linear random mixed-effect model was applied to compare the trend of changes in indexes between tPRK and conventional PRK methods, and an unstructured correlation matrix was used to evaluate the correlation between the fellow eyes and follow-up times. An intention-to-treat approach was adopted for analysis. *P* values less than 0.05 were considered significant.

## Results

**Fig. 1:**
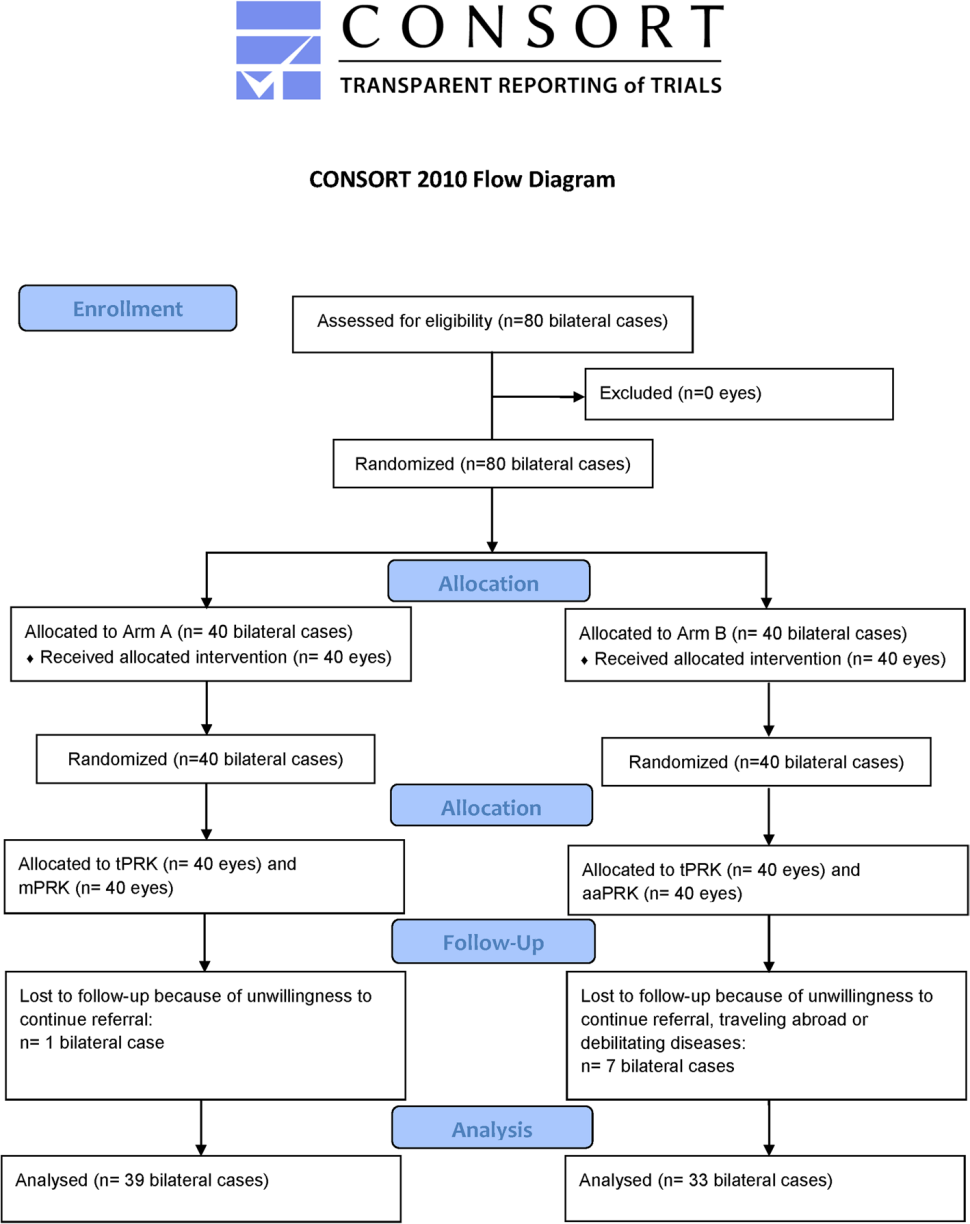
Flow diagram of the study (*n* = total subjects)

The trial was conducted from 6 May to 22 October, 2020. Eighty persons were included in the trial and randomly allocated to arms A or B. After excluding patients lost to follow-up during the first postoperative week, arm A included 39 bilateral patients with a mean age of 27.15 ± 5.60 years (range: 19 to 47) who underwent tPRK in one eye [21 cases (53.8%) in right eye] and mPRK in the fellow eye [18 cases (46.2%) in right eye]. Arm B included 33 patients with a mean age of 29.15 ± 7.44 years (19 to 49) who underwent tPRK in one eye [14 cases (42.4%) in right eye] and aaPRK in the fellow eye [19 cases (57.6%) in right eye]. Ten patients (25.6%) in arm A, and 13 subjects (39.4%) in arm B were male. In both arms A and B, 2 patients were lost to the 3-month follow-up. Figure 1 presents the flow diagram of the subjects from enrollment to data analysis.

Baseline characteristics of the participants in both arms are presented in Table 1. In both arms, mean UDVA was significantly lower in the tPRK group compared to the other groups (mPRK and aaPRK) (both *P* ≤ 0.03). In arm A, mean spherical error was significantly higher in the tPRK group (*P* = 0.01), and mean refractive cylinder error was significantly higher in the mPRK group (*P* = 0.05), but there was no significant difference in mean spherical equivalent between the tPRK and mPRK groups (*P* = 0.08).

**Table 1:** Baseline and 3-month postoperative parameters in arm A (*n* = 39 bilateral cases) and arm B (*n* = 33 bilateral cases)

		Arm A			Arm B		
		tPRK	mPRK	*P*-value*	tPRK	aaPRK	*P*-value*
UDVA (logMAR)	Baseline	1.37 ± 0.35	1.33 ± 0.36	0.03	1.46 ± 0.31	1.39 ± 0.33	0.008
	Postoperative	0.02 ± 0.04	0.02 ± 0.04	0.71	0.02 ± 0.04	0.01 ± 0.02	0.72
BCVA (logMAR)	Baseline	0.001 ± 0.001	0.003 ± 0.016	0.31	0.00 ± 0.01	0.00 ± 0.00	0.33
	Postoperative	0.01 ± 0.04	0.02 ± 0.03	0.17	0.01 ± 0.02	0.00 ± 0.00	0.80
SE (D)	Baseline	−4.52 ± 1.26	−4.36 ± 1.31	0.08	−4.55 ± 1.14	−4.33 ± 1.16	0.14
	Postoperative	0.27 ± 0.42	0.29 ± 0.37	0.67	0.33 ± 0.47	0.26 ± 0.36	0.27
Sphere (D)	Baseline	−4.02 ± 1.16	−3.78 ± 1.24	0.01	−4.03 ± 1.17	−3.76 ± 1.10	0.06
	Postoperative	0.47 ± 0.39	0.47 ± 0.38	0.92	0.52 ± 0.48	0.49 ± 0.34	0.91
Cylinder (D)	Baseline	−1.01 ± 0.77	−1.15 ± 0.77	0.05	−1.03 ± 0.82	−1.14 ± 0.74	0.30
	Postoperative	−0.41 ± 0.30	−0.33 ± 0.20	0.13	−0.36 ± 0.29	−0.48 ± 0.32	0.07

*tPRK* Transepithelial photorefractive keratectomy, *mPRK* Mechanical PRK, *aaPRK* Alcohol-assisted PRK, *UDVA* Uncorrected distance visual acuity, *CDVA* Corrected distance visual acuity, *SE* Spherical equivalent, *D* Diopter

*Paired t test

Data are presented as Mean ± SD

### Epithelial removal time

In both arms A and B, mean epithelial removal time in tPRK (13.00 ± 0.00) was significantly shorter compared to mPRK (30.23 ± 6.38) and aaPRK (57.12 ± 11.01) (both *P*<0.001).

### Symptoms

On the first postoperative day, patients experienced more pain, foreign body sensation, and burning sensation in the eye that had received tPRK compared to the eye treated with mPRK in arm A (*P* = 0.003, *P*<0.001, *P* = 0.003) or the eye treated with aaPRK in arm B (*P*<0.001, *P* = 0.004, *P*<0.001). However, there was no significant difference in other symptoms (all *P* > 0.05). There was no significant difference in the severity of symptoms between tPRK and mPRK in arm A or aaPRK in arm B on the third and seventh postoperative days (all *P* > 0.05) (Table 2).

**Table 2:** Postoperative symptoms in arm A (*n* = 39 bilateral cases) and arm B (*n* = 33 bilateral cases)

	Day	Method	Pain	FBS	Burning	Photophobia	Tearing	Dry eye
Arm A	1	tPRK	4.18 ± 3.68^*^	5.38 ± 3.64^**^	4.54 ± 3.32^*^	6.67 ± 3.40	5.77 ± 3.76	3.13 ± 3.30
		mPRK	2.49 ± 3.15	2.77 ± 3.23	3.31 ± 3.14	6.31 ± 3.51	5.10 ± 3.63	2.85 ± 3.00
	3	tPRK	2.21 ± 2.34	3.21 ± 3.00	2.90 ± 3.11	5.15 ± 3.00	3.08 ± 2.64	2.56 ± 2.76
		mPRK	1.85 ± 2.13	2.85 ± 2.67	2.90 ± 3.08	5.08 ± 2.97	2.79 ± 2.53	2.62 ± 2.65
	7	tPRK	0.37 ± 0.83	0.58 ± 1.07	0.37 ± 0.68	1.68 ± 1.70	0.16 ± 0.50	2.83 ± 1.98
		mPRK	0.53 ± 1.43	0.79 ± 1.55	0.26 ± 0.65	1.68 ± 1.77	0.26 ± 0.65	3.06 ± 2.01
Arm B	1	tPRK	5.52 ± 3.89^**^	5.94 ± 3.61^*^	5.73 ± 3.92^**^	5.30 ± 3.60	5.09 ± 3.20	3.61 ± 3.56
		aaPRK	3.21 ± 3.18	4.27 ± 3.44	3.91 ± 3.31	5.18 ± 3.55	4.73 ± 3.40	3.39 ± 3.40
	3	tPRK	3.45 ± 3.12	3.82 ± 3.32	2.97 ± 2.87	5.21 ± 3.26	3.18 ± 3.12	3.88 ± 3.42
		aaPRK	3.24 ± 2.67	3.94 ± 2.91	3.15 ± 2.88	5.33 ± 3.13	3.09 ± 3.06	3.91 ± 3.37
	7	tPRK	0.64 ± 1.74	0.71 ± 1.64	0.86 ± 1.75	2.79 ± 2.97	0.36 ± 1.34	3.93 ± 2.06
		aaPRK	0.50 ± 1.40	1.21 ± 2.64	1.79 ± 2.69	3.36 ± 3.15	0.86 ± 1.99	3.93 ± 2.02

*FBS* Foreign body sensation, *tPRK* Transepithelial photorefractive keratectomy, *mPRK* Mechanical PRK, *aaPRK* Alcohol-assisted PRK

Data are presented as Mean ± SD

Bold values are statistically significant

*P<0.01

**P<0.001

### CED, epithelial healing day, and epithelial healing rate

**Fig. 2:**
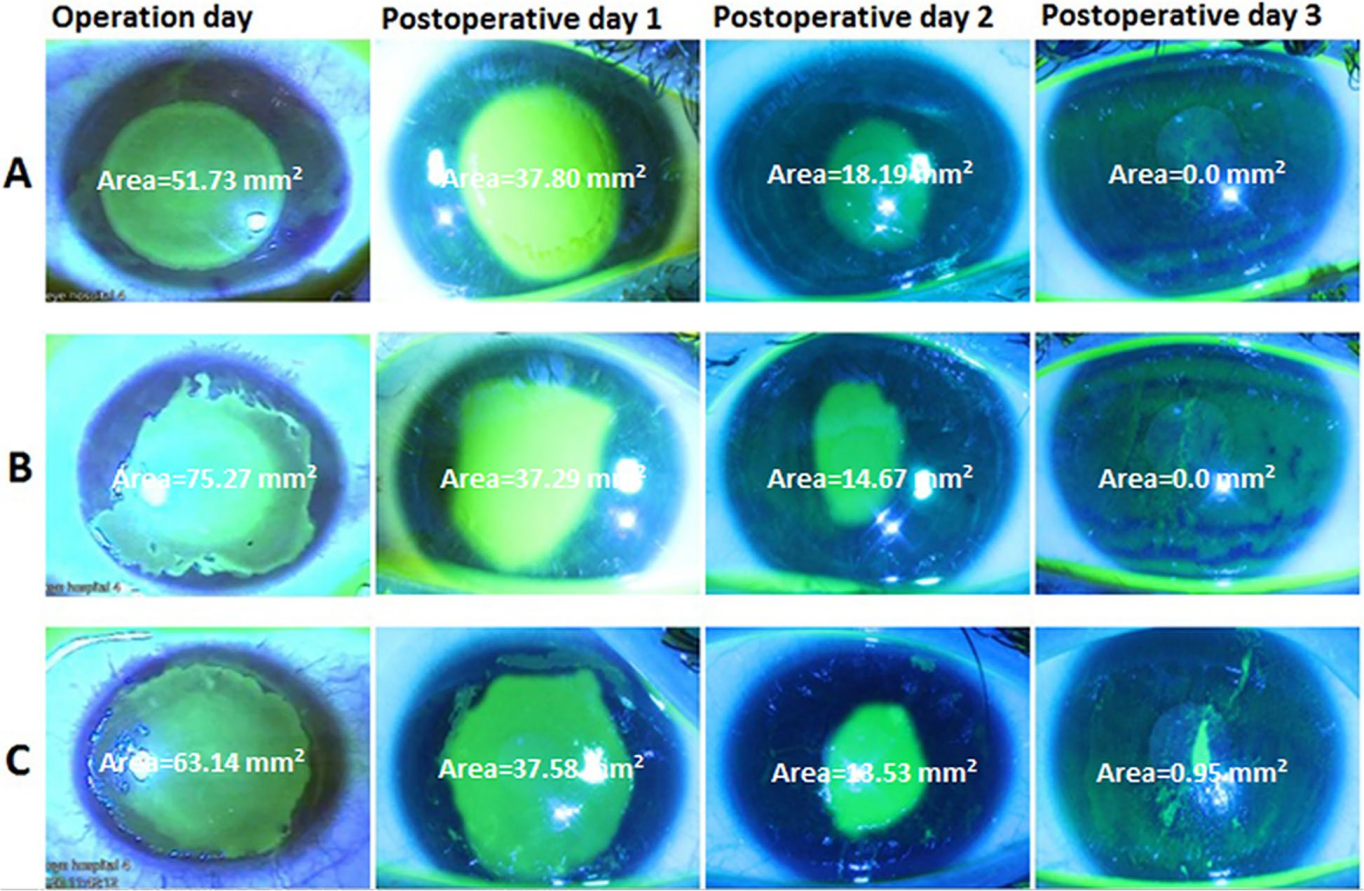
The images of corneal epithelial defect (CED) on operation day, and postoperative day 1, 2, and 3 in **A** an eye after transepithelial PRK (tPRK), **B **an eye after mechanical PRK (mPRK), and **C **an eye after alcohol-assisted PRK (aaPRK). The CED area in each image is calculated using the ImageJ software

**Fig. 3:**
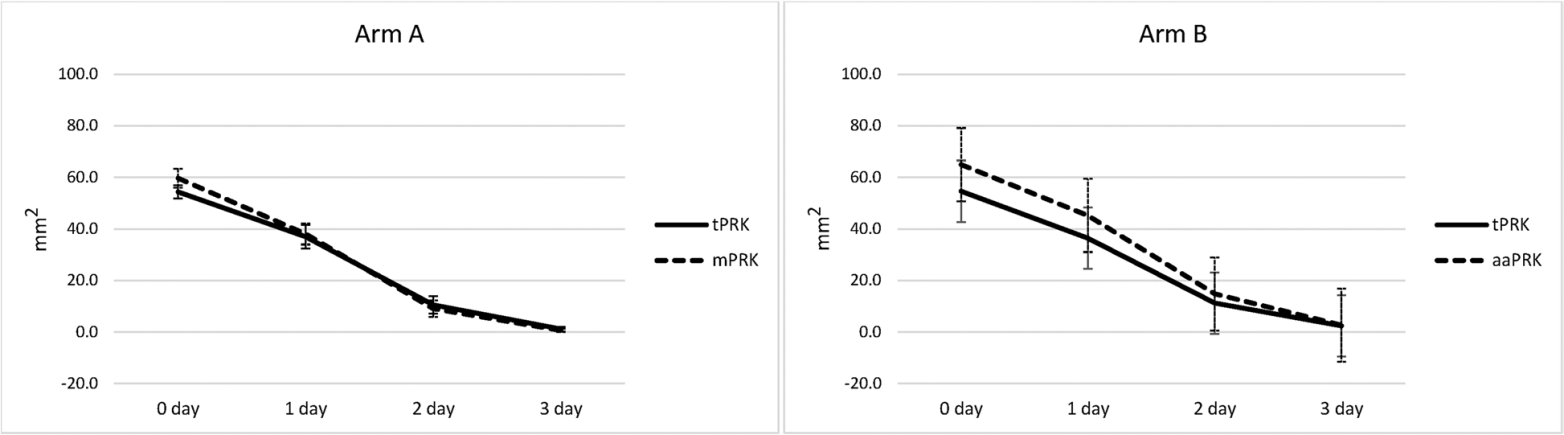
Corneal epithelial defect area (mm^2^) in arm A (transepithelial PRK (tPRK) / mechanical PRK (mPRK)) and arm B (tPRK / alcohol assisted PRK (aaPRK))

The CED healing process in the first three postoperative days is shown in Fig. 2. Table 3 and Fig. 3 compare the mean CED area on the operation day and the first three postoperative days in different groups.

**Table 3:** Corneal epithelial defect (CED) area in arm A (*n* = 39 bilateral cases) and arm B (*n* = 33 bilateral cases)

	Method	Operation day	Day 1	Day 2	Day 3	3-day change	***P***-value^**†**^
**Arm A**	tPRK	54.35 ± 5.26	36.97 ± 9.29	10.55 ± 6.90	1.17 ± 1.85	−55.60 ± 5.78	0.02
	mPRK	59.68 ± 7.34^*^	38.02 ± 8.14	9.06 ± 6.29	0.61 ± 1.32	−61.68 ± 7.61	
**Arm B**	tPRK	54.27 ± 4.76	37.34 ± 13.07	11.60 ± 9.10	2.28 ± 4.36	−53.77 ± 7.10	0.16
	aaPRK	64.96 ± 6.54^**^	45.23 ± 7.36^*^	14.80 ± 6.29	2.67 ± 3.82	−61.27 ± 7.12	

*tPRK* Transepithelial photorefractive keratectomy, *mPRK* Mechanical PRK, *aaPRK* Alcohol assisted PRK

^**†**^Based on three-day change of CED by random mixed-effect model adjusted for correlation of fellow eyes and follow-up times

All data are presented as Mean ± SD (mm^2^)

Bold values are statistically significant by random mixed-effect model adjusted for correlation of fellow eyes

*P<0.01

**P<0.001

In arm A, mean CED area was significantly smaller in the tPRK group compared to the mPRK group only on the operation day (*P* = 0.003) with no significant difference on the following days (all *P* > 0.05). The three-day decrease in CED area was significantly greater in the mPRK group versus the tPRK group (61.68 ± 7.61 vs. 55.60 ± 5.78 mm^2^, *P* = 0.02). There was no significant difference in the mean epithelial healing time between tPRK (3.74 ± 0.82 days) and mPRK (3.59 ± 0.79 days) (*P* = 0.21).

In arm B, mean CED area was significantly smaller in the tPRK group compared to the aaPRK group on the operation day (*P* < 0.001) and the first postoperative day (*P* = 0.004). The three-day decrease in CED area was not significantly different between the two methods (*P* = 0.16). There was no significant difference in the mean epithelial healing time between tPRK (3.67 ± 0.92 days) and aaPRK (3.67 ± 0.74 days) (*P* = 1.00).

The epithelial healing rate was calculated in mm^2^/day according to the change in CED area between two consecutive days. In both arms, the healing rate was faster with the conventional methods compared to tPRK (both *P* < 0.001). Based on daily comparisons in arm A, the healing rate was significantly higher in mPRK compared to tPRK only on the first postoperative day (*P* = 0.04). In arm B, the healing rate was faster in aaPRK compared to tPRK on the second (*P* = 0.01) and third (*P* = 0.04) postoperative days (Table 4).

**Table 4:** Healing rate in arm A (*n* = 39 bilateral cases) and arm B (*n* = 33 bilateral cases)

	Method	Day 0 to 1	Day 1 to 2	Day 2 to 3	***P***-value^**†**^
**Arm A**	tPRK	17.38 ± 7.63	26.42 ± 6.35	9.38 ± 6.02	<0.001
	mPRK	21.66 ± 9.35^*^	28.96 ± 6.89	8.45 ± 5.27	
**Arm B**	tPRK	16.94 ± 12.93	25.74 ± 11.21	9.32 ± 6.25	<0.001
	aaPRK	19.73 ± 8.75	30.43 ± 8.06^**^	12.13 ± 4.80^*^	

*tPRK* Transepithelial photorefractive keratectomy, *mPRK* Mechanical PRK, *aaPRK* Alcohol assisted PRK

^**†**^Based on three-day healing rate by random mixed-effect model adjusted for correlation of fellow eyes and follow-up times

All data are presented as Mean ± SD (mm^2^/day)

Bold values are statistically significant by random mixed-effect model adjusted for correlation of fellow eyes

*P<0.05

**P<0.01

### Safety, efficacy, and spherical equivalent refractive accuracy

At three months after surgery, 34 eyes (91.9%) had a UDVA of 20/20 or better in both groups in arm A. In arm B, 27 eyes (87.5%) in the tPRK group and 30 eyes (96.9%) in the aaPRK group had a UDVA of 20/20 or better.

**Fig. 4:**
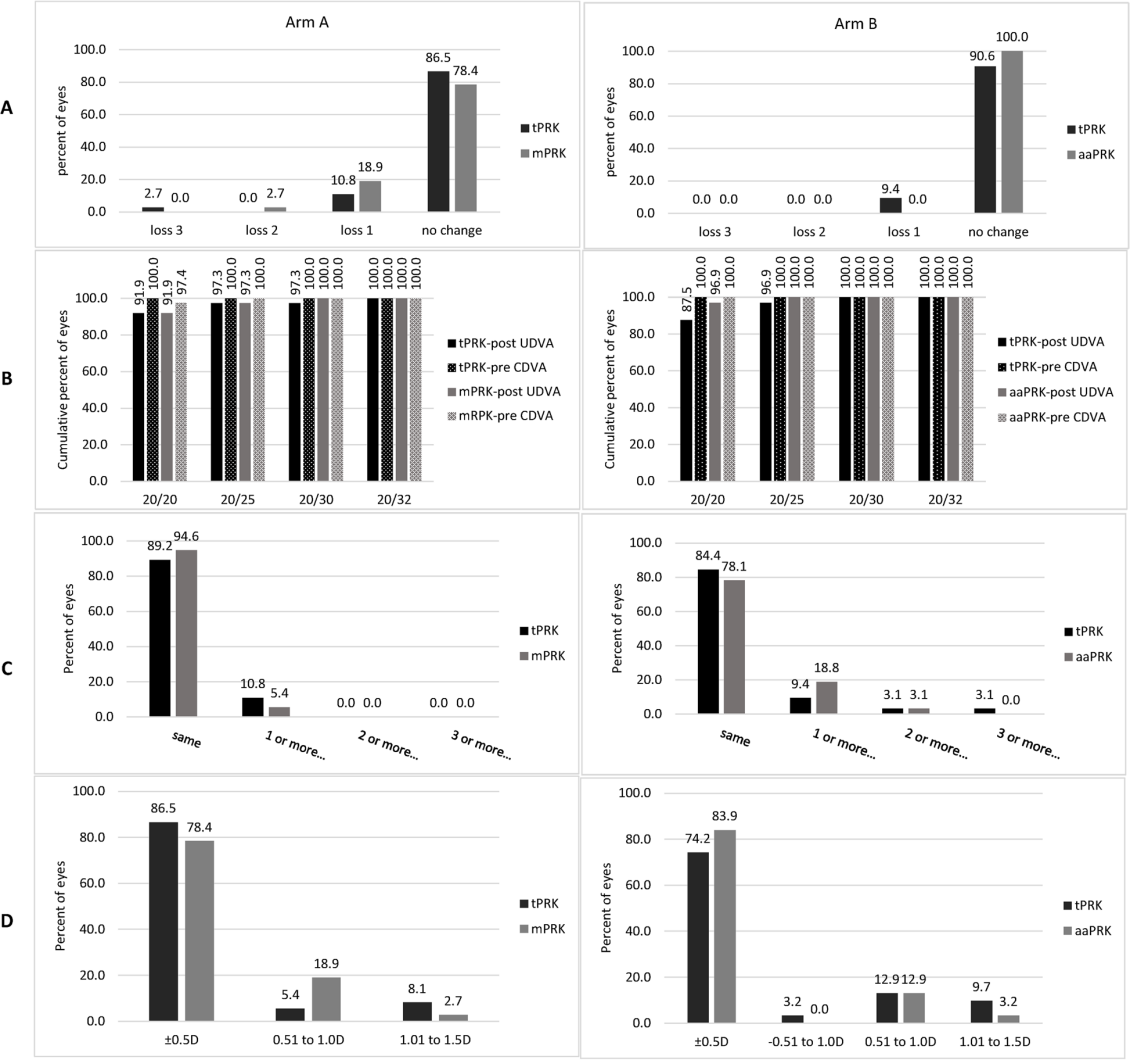
Three-month safety, efficacy and spherical equivalent refractive accuracy after transepithelial PRK (tPRK), mechanical PRK (mPRK), and alcohol-assisted PRK (aaPRK), **A** Gain and loss of corrected distance visual acuity (CDVA), **B** Cumulative Snellen visual acuity (20/* or better), **C** Difference between postoperative uncorrected distance visual acuity (UDVA) and preoperative CDVA (Snellen lines), **D** accuracy of spherical equivalent refraction to intended target

There was no significant difference in the safety index between tPRK and mPRK in arm A (0.99 ± 0.03 vs. 0.98 ± 0.05, *P* = 0.50) or aaPRK in arm B (1.00 ± 0.00 vs. 0.99 ± 0.03, *P* = 0.68). No significant difference was found in the efficacy index between tPRK and mPRK in arm A (0.98 ± 0.05 vs. 0.97 ± 0.05, *P* = 0.98) or aaPRK in arm B (0.96 ± 0.07 vs. 0.97 ± 0.05, *P* = 0.88). Figure 4(A, B and C) shows the safety and efficacy indexes in each arm.

At three months after surgery, mean postoperative spherical equivalent had decreased in both groups in both arms (all *P* < 0.001). Figure 4(D) shows the achieved spherical equivalent compared to the intended target in each arm. In arm A, 32 eyes (86.5%) in the tPRK group and 29 eyes (78.4%) in the mPRK group had a spherical equivalent within ±0.50 D of the intended target (*P* = 0.20). In arm B, 23 eyes (74.2%) in the tPRK group and 26 eyes (83.9%) in the aaPRK group were within ±0.50 D of the intended target 3 months after surgery (*P* = 0.63).

### Postoperative corneal haze

At three months after surgery, 3 cases in arm A (7.69%) and 2 cases in arm B (6.06%) had grade 0.5 or 1 corneal haze in one (one tPRK eye and one mPRK eye in arm A) or both eyes (one case in arm A and two cases in arm B). The differences in corneal haze severity between tPRK and mPRK in arm A (0.03 ± 0.11 vs. 0.05 ± 0.23, *P* = 0.31) or tPRK and aaPRK in arm B (0.05 ± 0.20 vs. 0.05 ± 0.20, *P* = 1.00) were not statistically significant.

## Discussion

In this study, the epithelial healing rate and the clinical outcomes of tPRK were compared to conventional PRK methods (mPRK and aaPRK). The results showed that all three methods were effective in terms of refractive and visual outcomes, and they were similar in terms of the time to complete re-epithelialization, however, the rate of epithelial healing was faster with conventional PRK methods considering the initial CED area. Moreover, patients experienced less pain and other inconvenient symptoms in conventional PRK methods compared to tPRK in the first postoperative day.

Faster epithelial healing after PRK speeds visual recovery, and decreases the period of postoperative pain and discomfort, and the risk of adverse events. Therefore, many researchers have tried to find methods to accelerate the post-PRK re-epithelialization process. Several studies evaluated the usage of pharmacological agents including topical Plasma Rich in Growth Factors (PRGF) [19, 20], basic Fibroblast Growth Factor [21, 22], Cytochrome c Peroxidase [22–24], and Polydeoxyribonucleotide (PDRN) [25], oral l-Cysteine [21, 26, 27], Omega-3 [28], and high dose Vitamin A + Vitamin E [29] supplements. Others have studied the choice of postoperative antibiotic [30, 31] and bandage contact lens [32–34]. In the present study, we compared the effect of different epithelial debridement methods on epithelial healing acceleration.

To the best of our knowledge, this is the first clinical trial for simultaneous comparison of two popular conventional PRK methods (mPRK and aaPRK) with the newest laser ablation method, i.e. tPRK. To control the effect of surgeon’s experience, all operations were performed by the same ophthalmologist. The study had a contralateral design to ensure homogenous preoperative features in conventional and tPRK groups. All operations were done using the Amaris excimer laser and with a similar optical zone. The main difference between these procedures was the method of epithelial removal, which was done simultaneously with laser refractive ablation in tPRK causing an epithelial wound with regular margins; therefore, the epithelial removal area was equal to the stromal ablation area. Decreased surgical time in PRK reduces the chance of stromal dehydration and improves control over the patient’s fixation during surgery. In conventional PRK methods, the surgical time is largely surgeon-dependent. In tPRK, the surgical time is determined automatically by the laser machine according to the amount of required ablation. Previous studies that compared the total surgical time between tPRK and conventional PRK methods found that tPRK was faster than mPRK and aaPRK [11, 12, 15]. In the present study, instead of total surgical time, we compared the epithelial removal time because we expected a similar stromal ablation time in all three methods. The results showed that the epithelial removal time was significantly shorter in tPRK compared to both mPRK and aaPRK.

The results of the present study showed a similar re-epithelialization time (3.59–3.74 days) with all three methods. However, considering the initial CED area, the epithelial healing rate was faster on the first postoperative day with mPRK compared to tPRK in arm A. This may be explained by the difference in the epithelial removal surface area and the stromal ablation area between two groups. In tPRK, the epithelial removal area and the stromal ablation area are equal while the epithelial removal area is larger than the stromal ablation area with mPRK which could alter the CED healing rate [9, 14]. In other words, the re-epithelialization rate may have been faster in the areas without stromal ablation, resulting in a seemingly accelerated healing overall on the first postoperative day with mPRK. There was no significant difference between tPRK and mPRK in subsequent days when the CED area was limited to the stromal ablation area. In arm B, although tPRK and aaPRK had a similar epithelial healing rate on the first postoperative day, aaPRK showed a faster healing rate on the second and third postoperative days. This discrepancy between aaPRK and mPRK may be related to the transitory toxic effects of alcohol on limbal stem cells or remaining epithelial cells, which, unlike the mPRK method, resulted in a slower epithelial healing rate after aaPRK and a similar healing rate on the first postoperative day in tPRK and aaPRK methods [9]. Our findings do not agree with previous studies reporting a faster epithelial healing rate with tPRK compared to aaPRK and mPRK [11, 12, 14, 15, 17]. This may be due to methodological differences in CED size calculation and monitoring. In the present study, CED images were captured on the operation day and following days and the CED area was calculated using an image processing software. However, in the above mentioned studies, the CED size was subjectively measured using a slit lamp, mostly in one meridian (horizontal diameter). In addition, the initial size of the CED was ignored in most studies.

Postoperative pain and discomfort are the most prominent disadvantages of PRK. Most of the previous studies reported less postoperative pain in tPRK compared to mPRK and aaPRK [12, 14, 15, 17]. However, Zarei-Ghanavati et al. [11] conducted a contralateral study on myopic and myopic astigmatism patients undergoing tPRK and mPRK and reported more pain, photophobia, and tearing on days 1, 3, and 7 postoperatively in the tPRK group. In the present study, pain, foreign body sensation, and burning sensation were more intense in the tPRK eyes compared to conventional PRK eyes in the first 24 h after surgery; however, no difference was found between tPRK and conventional PRK on days 3 and 7 postoperatively. In addition, the severity of photophobia, dry eye, and tearing were similar in all groups on all postoperative days. The exact reason for increased pain after tPRK is not clear, but it is believed to be related to excessive stimulation of polymodal nociceptors in tPRK; they comprise 70% of the corneal nerve fibers and are sensitive to heat in addition to mechanical stimuli [11, 35]. These fibers start firing at temperatures above 30–40 °C [35]. The use of laser during PRK increases the temperature to 53 °C in the stromal bed, resulting in a burning sensation and sharp pain due to the stimulation of nociceptors [36]. In tPRK, the cornea is subjected to an additional 13 s of stimulation, which may result in increased temperature and further stimulation of nociceptors. Cool BSS irrigation before and after ablation is typically recommended with tPRK to avoid this additional stimulation [37, 38]. In the present study, since we aimed to compare the pure effect of epithelial removal methods, we tried to keep all other influential factors the same; and thus, we used the same BSS temperature (room temperature) in all surgeries. Our results showed that the effect of additional stimulations on tPRK did not appear to continue beyond the first 24 h after surgery.

There was no difference in UDVA, CDVA, refractive error, or spherical equivalent refractive accuracy between tPRK, mPRK, and aaPRK three months after the operation. The safety and efficacy indexes of all three methods were above 95%. These findings were consistent with previous studies that found no difference in refractive and visual outcomes between tPRK and conventional PRK methods 3, 6, and 12 months after the operation [10–13, 15, 16, 39]. However, Naderi et al. [14] suggested that tPRK was superior to aaPRK in terms of visual recovery two months after surgery. Moreover, the safety, efficacy, and refractive outcomes were better in patients receiving tPRK.

Corneal haze is a well-known side effect of PRK. Aslanides et al. [17] and Bakhsh et al. [12] compared tPRK and aaPRK and found a lower incidence of haze in the tPRK method in the three-month follow-up. However, the incidence of haze was similar in tPRK and conventional PRK methods in the present study, which was consistent with the results of studies conducted by Ghobashy et al. [16] and Kaluzny et al. [13]

One limitation of this study may be the use of MMC, primarily to prevent the development of stromal haze, which may affect epithelial regeneration [40]. We used MMC in all three surgical groups, and the protocol for using MMC was the same for all surgeries. However, the results of our study may only be generalizable to patients undergoing PRK + MMC. Another limitation of the study was the daily replacement of the bandage contact lens to photograph the CED area because it was not possible to stain the cornea with the bandage contact lens in place. This may have interfered with normal healing and delayed the process to some extent. However, bandage contact lens replacement was similar in both conventional PRK and tPRK eyes, and they were replaced simultaneously. In addition, in a recent study, AlDahash et al. [41] showed that lens replacement on the first postoperative day, when most epithelial regeneration occurs, has no effect on the healing process.

## Conclusions

The three-month visual and refractive outcomes of tPRK, mPRK, and aaPRK were similar in the present study. However, the patients experienced less pain and discomfort in the first 24 h after surgery with conventional PRK methods. Furthermore, although it seems that the epithelial healing rate is faster in conventional PRK methods compared to tPRK, the mean complete re-epithelialization time was similar in all methods due to the small area of the initial CED in tPRK. The results of this study may be important for the selection of PRK method in patients who are more sensitive to pain.

## Data Availability

The datasets used and/or analyzed during the current study are available from the corresponding author on reasonable request.

## Abbreviations

PRK: Photorefractive keratectomy
mPRK: Mechanical epithelial debridement photorefractive keratectomy
aaPRK: Alcohol-assisted photorefractive keratectomy
tPRK: Transepithelial photorefractive keratectomy
CED: Corneal epithelial defect
D: Diopters
BSS: Balanced saline solution
OZ: Optical zone
GEE: Generalized estimating equations.
